# Comprehensive Hip Fracture Care Program: Successive Implementation in 3 Hospitals

**DOI:** 10.1177/2151459319846057

**Published:** 2019-05-15

**Authors:** Kelly Jackson, Mary Bachhuber, Dawn Bowden, Katherine Etter, Cindy Tong

**Affiliations:** 1 Neuroscience Service Line, HonorHealth Osborn Medical Campus Administration, HonorHealth System, Scottsdale, AZ, USA; 2 Orthopedics, HonorHealth System, Scottsdale, AZ, USA; 3 Health Economics & Market Access, Johnson & Johnson, Highlands Ranch, CO, USA; 4 Healthcare Analytics, Health Economics & Market Access, Johnson & Johnson, Raynham, MA, USA; 5 Health Economics & Market Access Analytics, Johnson & Johnson, Bridgewater, NJ, USA

**Keywords:** hip fracture, femoral fracture, integrated delivery of health care, patient care team, quality assurance

## Abstract

**Introduction:**

Hip fractures are common and costly in the elderly population, often contributing to loss of function and independence. Prompt, coordinated surgical care may improve clinical and economic outcomes for this population.

**Materials and Methods:**

We created an interdisciplinary care program focused on minimizing time spent immobilized awaiting surgery and streamlining the care pathway for hip fracture. Patients older than 65 years with any hip fracture type including hip fracture repair Diagnosis-Related Group codes (MS-DRG 480, 481, or 482) and MS-DRG 469 and 470 with a hip fracture diagnosis were included in the study. The Hip Fracture Care program (HFCP) was implemented on a staggered basis in 3 hospitals in the HonorHealth system. Time to surgery, length of stay, and discharge location (home/skilled nursing facility) were compared pre- and post-intervention, utilizing an interrupted time series analysis to account for background trends.

**Results:**

More than 2000 patients across the 3 facilities received HFCP care; demographics were similar for the 826 patients serving as the pre-implementation comparison group. Mean (standard deviation [SD]) length of stay decreased from 5.6 (4.0) to 4.7 (2.9) days (mean difference 0.9 days; *P* < .05). Mean (SD) time from admission to the operating room decreased from 30.8 (21.1) to 25.6 (20.5) hours (mean difference 5.2 hours; *P* < .05). There was no change in the proportion of patients discharged to home versus skilled nursing facility.

**Discussion:**

Optimal care of this vulnerable population can significantly reduce the time to surgery and length of stay.

**Conclusions:**

Length of stay was reduced by nearly 1 day with implementation of a multifactorial program for hip fracture care.

## Introduction

Hip fractures are a significant public health concern, affecting nearly 300,000 people in the United States each year.^1^ Hip fractures contribute disproportionately to the overall cost of osteoporosis-related fracture, accounting for 72% of costs while only representing 14% of all fractures.^1^ The prognosis for older adults after fracture is poor: fewer than half of patients regain function at 1 year, and they face significantly increased risk of death or institutionalization within 2 years of fracture.^2,3^ In addition to increasing age, female sex predicts greater risk of adverse events and discharge to nonhome care facilities after hip fracture surgery.^4^ Patients who receive prompt surgical care experience reduced mortality and complications, suggesting that interventions designed to streamline time to surgery may improve clinical and economic outcomes for both patients and institutions.^5^ Surgery within 24 to 48 hours has been identified as one of the components of quality care in hip fracture by the Agency for Healthcare Research and Quality, with the benefit most pronounced for frail patients.^6^ Organized, interdisciplinary fracture programs have in some cases successfully reduced patient morbidity, shortened length of stay (LOS), and improved functional outcomes, but evidence for this approach is primarily limited to single-center interventions.^7-13^

The purpose of the current study was to evaluate whether implementing an interdisciplinary Hip Fracture Care program (HFCP) reduces the economic burden and improves patient care among patients treated in a multihospital health system.

## Materials and Methods

### Patients

Patients older than 65 years who were admitted to 1 of 3 HonorHealth hospitals between January 2011 and December 2016 were the population of interest. The HonorHealth health system identified an opportunity to improve care for this vulnerable population, and in particular was motivated by concern for complications of immobility as patients awaited surgery. The health system consists of 5 magnet-recognized facilities in the greater Phoenix, Arizona area, 3 of which participated in the current study: Osborn Medical Center (337 beds; level I trauma center), Shea Medical Center (433 beds), and Thompson Peak Medical Center (120 beds). The HFCP was implemented beginning in January 2012 at Osborn, followed by Shea (September 2012) and Thompson Peak (February 2013). The study was conducted as part of the hospital system’s quality improvement initiative and was therefore considered exempt from the institutional review board review requirement.

Our study used a pre-and post-design based on the staggered implementation date of the HFCP at each of the 3 hospitals. Data were obtained via retrospective review of deidentified records collected from the HonorHealth electronic health records for patients with any hip fracture type including hip fracture repair Diagnosis-Related Group codes (MS-DRG 480, 481 or 482) and MS-DRG 469 and 470 with a hip fracture diagnosis.

### Intervention

**Figure 1.**
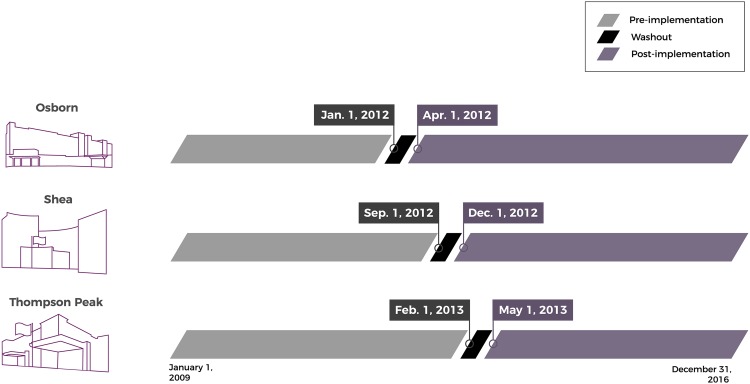
Hip fracture care program implementation timeline.

The HFCP was developed in consultation with key stakeholders and implemented for all eligible patients in each of the 3 hospitals sequentially, beginning in 2012 (Figure 1). The HFCP consisted of a standardized team-based approach designed to get most patients into surgery within 48 hours of fracture or sooner, as well as to optimize the treatment plan from admission through discharge. Key components of the HFCP included addition of a geriatric fracture nurse practitioner, staff education, a care bundle including pressure-reducing surfaces, avoidance of skeletal traction, antibiotic and antithrombotic prophylaxis, and early mobilization.

The care team consisted of a nurse practitioner, 2 orthopedic surgeons, other physician subspecialties, the anesthesia team, administration, physical and occupational therapists, nursing/case management, and the quality department. Adherence to the care bundle was tracked and a report was distributed quarterly to the care team and hospital administration for benchmarking purposes.

### Primary and Secondary Outcomes

The primary outcome was mean hospital LOS, including total LOS and post-operative LOS. Secondary outcomes included discharge status (percent home vs skilled nursing facility [SNF]/other) and time to surgery. The published standard for time to surgery is calculated from the time of admission until arrival in the operating room (OR).^14^ For this study, time to surgery was calculated either as time from admission to OR or emergency department (ED) to OR for patients admitted through the ED.

### Analyses

The study was designed as a pre- and post-comparison with an interrupted time series analysis. Also known as segmented regression analysis, interrupted time series analysis controls for trends in outcomes that may be attributable to external factors preceding a change in practice.^15^ A sample size of 1,291 was calculated using a one-sided *t* test as sufficient to detect a modest (5%) reduction in the primary end point of LOS with 80% power. The following data elements were captured for all patients: facility, age, sex, marital status, ethnicity, principal diagnosis, principal procedure, DRGs, payor, admission status (emergency, elective, trauma center, urgent), and admission location (through ED: yes/no).^16^ Hospital room and board costs were estimated on a per-patient, per-day basis for each of the 3 sites using unit costs for routine bed days estimated from each hospital’s 2016 Medicare cost report.

Continuous data were summarized using counts, medians, means, ranges, and standard deviations (SD), while categorical data were summarized using frequencies and percentages. Differences between the pre- and post-implementation groups were analyzed using interrupted time series analyses for the primary (LOS) and secondary outcomes (discharge location and time to surgery from admission/ED). Interrupted time series analysis was used to evaluate whether the program had an effect significantly greater than the underlying time trend.^17^ Mean values for each quarterly time period were used to build the models, instead of using individual data points, to reduce variation. Multivariate models were used to adjust for potential differences in the mix of primary procedures during the pre- and post-implementation periods. The percentage of patients with each type of procedure at each time point were used as covariates in the model.

A *P* value of less than .05 was regarded as statistically significant. Data were analyzed using R Studio version 1.1.4 (RStudio Inc, Boston, Massachusetts) and SAS Enterprise Guide version 7.1 (SAS Institute, Inc, Cary, North Carolina).

## Results

### Patient Characteristics

**Figure 2.**
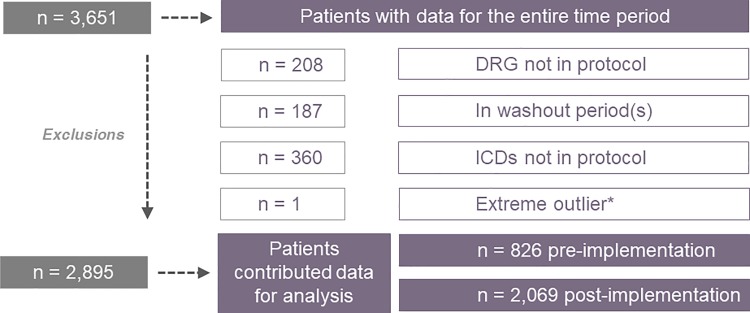
Patient attrition. *Admission time to OR time assumed to be erroneous (>1000 days). DRG indicates diagnosis-related group; ICD, international classification of disease; OR, operating room.

Records for 3651 patients were reviewed for eligibility, including both pre- and post-implementation periods. Records between the pre- and post-implementation periods were defined as the washout period, to allow adjustment of processes before assessing the impact of the program. After ineligible and washout records were removed, a review of the remaining 2896 records revealed 1 patient with an extreme outlier value for time to surgery (Figure 2). This entry was assumed to be erroneous and was not used in the analysis of end points. Therefore, a total of 2,895 patients were included in the analysis (826 prior to HFCP and 2,069 after full HFCP implementation at the respective site). Patients were elderly (mean age over 82 years) and predominantly female, as expected. The majority of patients were covered by Medicare, and 97% were Caucasian. Mean age, percentage of female patients, and source of admission did not differ between the pre- and post-implementation periods (Table 1). Slightly more patients in the post-HFCP period were married. The type of fracture and the procedure type differed statistically between the pre- and post-implementation periods, although these numeric differences were not large.

**Table 1.** Patient Characteristics: Pre- and Post-implementation of Hip Fracture Care Program.^a^

	Pre-implementation, (n = 826)	Post-implementation (n = 2069)
Age, years; mean (SD)	82.8 (7.6)	82.3 (7.1)
Female (%)	71.5	70.7
Marital status (%)^b^		
Married	31.5	38.8
Unmarried (including divorced, widowed)	68.3	60.4
Facility		
Osborn	431	872
Shea	299	882
Thompson peak	96	315
Admission status (%)^c^		
Emergency	95.2	93.8
Elective	0.6	0.8
Trauma center	1.1	1.4
Urgent	3.1	4.0
Type of health insurance (%)		
Medicare	63.9	67.1
Other/not specified	36.1	32.9
Procedure type (%)^b,d^		
Fixation	61.7	63.6
Hemiarthroplasty or other	38.3	36.4
Type of fracture (principal diagnosis)^b^		
Femoral neck or head	24.7	27.6
Peritrochanteric	52.5	53.5
Other^e^	22.8	18.9

Abbreviation: SD, standard deviation.

^a^Categories may not total to 100% due to rounding and/or missing data.

^b^ *P* < .05 for pre versus post difference.

^c^HonorHealth uses the Optum 360°, LLC. Uniform Billing Editor (2016) to determine patient status codes that are required for Medicare claims. Emergency is defined as: “The patient requires immediate medical intervention due to a severe, life threatening, or potentially disabling condition. Generally the patient is admitted through the ED.” Urgent is defined as: “The patient requires immediate attention for care and treatment of a physical or mental disorder. Generally the patient is admitted to the first available and suitable accommodation.”

^d^Principal procedure types included: Internal fixation-femur, closed reduction-internal fixation femur, open reduction-internal fixation femur, partial hip replacement, other-arthrotomy hip, partial ostectomy-femur.

^e^Other fracture types included but not limited to: intracapsular, intertrochanteric, midcervical, and pathological.

### Hip Fracture Care Program

**Figure 3.**
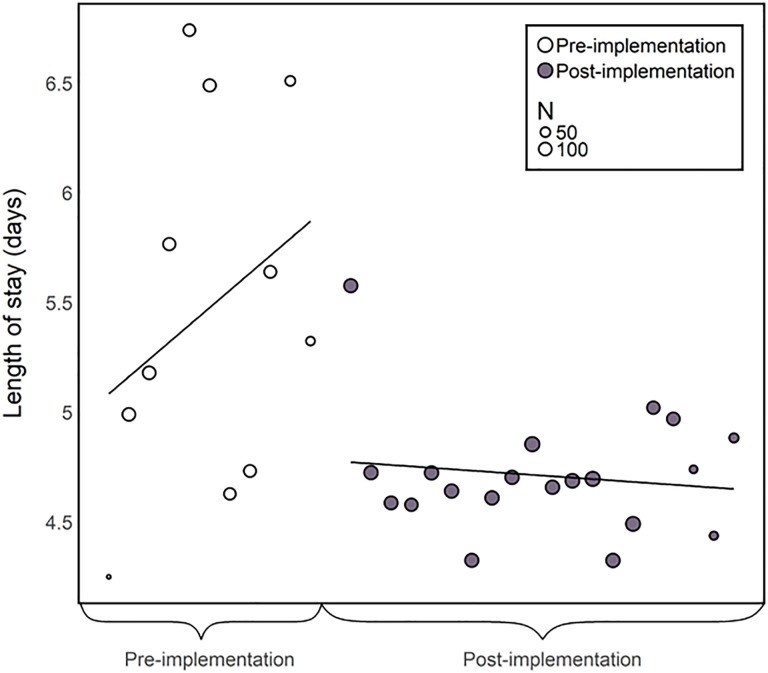
Mean length of stay.

The HFCP was successfully implemented across the 3 hospitals. Mean LOS decreased from 5.6 to 4.7 days after HFCP implementation (mean difference: 0.9 days). Mean (SD) time from admission to the OR decreased from 30.8 (21.1) to 25.6 (20.5) hours (mean difference: 5.2 hours). Mean (SD) ED to OR time decreased from 32.7 (22.1) to 29.4 (73.6) hours, but there was greater variability in the post-HFCP period and the difference did not reach statistical significance. The percentage of patients discharged to home was relatively stable between the pre- and post-implementation periods (Table 2). Differences in LOS and time from nonemergent admission to surgery were statistically significant based on the interrupted time series analysis. For LOS, the pre versus post difference also remained statistically significant after adjusting for procedure type (*P* = .046). The time series analysis revealed a sharp drop in LOS that was maintained for approximately 4 years after implementation of the HFCP (Figure 3). Mean per-patient hospital costs for room and board decreased by an estimated US$912 per hip fracture patient after implementation of the HFCP.

**Table 2.** Outcomes Pre- and Post-implementation of Hip Fracture Care Program.

	Pre-implementation (n = 826)	Post-implementation (n = 2,069)
LOS, days; mean (SD)^a^	5.6 (4.0)	4.7 (2.9)
Cost of LOS, US$; mean (SD)^a,b^	$6,083 (4340)	$5,171 (3,098)
Admission to OR, hours; mean (SD)^a^	30.8 (21.1)	25.6 (20.5)
ED to OR, hours; mean (SD)	32.7 (22.1)	29.4 (73.6)
Discharged to SNF or other than home, %	91.4	92.3

Abbreviations: ED, emergency department; LOS, length of stay; OR, operating room; SD, standard deviation; SNF, skilled nursing facility.

^a^ *P* < .05 for pre vs post difference by interrupted time series model.

^b^Cost basis per day: US$1086 Osborn, US$1073 Shea, US$1217 Thompson Peak, based on Medicare reimbursement.

## Discussion

Our evaluation of an interdisciplinary HFCP demonstrated that the program was associated with improvements in clinical metrics and economic outcomes. The program reduced overall LOS for hip fracture by nearly a day in our study population. The time series analysis suggests that this statistically significant decrease was maintained across study sites for approximately 4 years after the implementation. Long-term data on the persistence of gains after quality care interventions in the surgical setting is lacking, but evidence from other care improvement interventions applied at the hospital or health-system level suggest that initial positive impacts may plateau or decline over time.^18,19^

Mean LOS pre-implementation at the sites in this study was similar to that observed within the National Surgical Quality Improvement Program data (Basques 2015, 5.6 days), and slightly lower than that reported in 2 contemporary studies: Neuman 2014 (6.2 days, NY State data), Samuel 2016 (6.6 days, National Trauma Data Bank).^20-22^ After full implementation of the HFCP, the mean LOS was lower than that reported within several studies.^20-22^ The proportion of patients discharged to a SNF after surgery was higher than that reported in some published estimates, but in line with the 89% estimate obtained from Medicare data from 2009 to 2016.^23,24^ Delays in time from admission to surgery in excess of 48 hours are associated with increased risk of post-operative mortality.^25^ Some of the more common delays at the HonorHealth health system were related to unstable medical conditions that required medical management such as coagulopathy and clearance for cardiac comorbidities. The HFCP was associated with a significant reduction in the time from nonemergent admission to OR, and most patients entered surgery within the 48-hour window.

In addition to reducing risk for vulnerable elderly populations by avoiding unnecessary time in acute care facilities, quality improvement initiatives like the HFCP should be cost effective. Decreasing hospital LOS by an average of 1 day per patient is estimated to have reduced costs by US$340,936 at the HonorHealth health system for 2016 compared to the average cost of the pre-implementation period.

Demographic trends in the United States (aging of baby boomer generation) indicate an urgent need to address potential risk and improve outcomes in the hip fracture population. After a decline from 1995 to 2012, a recent analysis found age-adjusted rates of incident hip fracture in the United States have plateaued at levels higher than expected in 2012 through 2015.^26,27^ Widespread implementation of interdisciplinary care programs and other efforts spurred by value-based care and bundled payments may ameliorate the impact to health-care systems from this trend.

Strengths of this program include broad patient selection criteria, a standardized program to provide care, and a consistent assessment of the primary end point. Our sample size met the projected number of patients sufficient to detect an impact on the primary end point, LOS. In addition, unlike most previous reports of similar programs, the HFCP at HonorHealth was implemented in 3 separate hospitals at various time points, allowing assessment of the relevance of the program’s effect across hospitals within a health system. Age and sex for the study population are consistent with published demographics of the hip fracture population in the United States.^1, 20, 28^

Limitations include the lack of a randomized control group and the limited data on clinical variables. We experienced the typical challenges of quality improvement research, including the difficulty of defining a clear pre/post-period since quality improvement activities are continuous. We attempted to define a washout period specific to the HFCP to mitigate this issue, but nonetheless, the distinction between pre and post periods may be blurred by participation of some personnel at both “pre” and “post” rollout sites. Further, 97% of the patients in this study were Caucasian, which may limit generalizability.

Though implementation of the HFCP was initially focused on reducing time to the OR and decreasing hospital LOS, the hospital system has since focused on reducing the risk of hospital readmissions for this population. Because of the complicated relationship between LOS and readmission risk, collection and analysis of readmission data pre- and post-implementation of the HFCP is an area for future research. Other areas warranting investigation include avoidable complications—for example, hospital-acquired pressure ulcers—and patient-centered outcomes, including health-related quality of life and functional status.

## Conclusions

This study is a confirmatory study showcasing the distinctions and impacts of implementation across multiple sites across a hospital system. An interdisciplinary approach to hip fracture care in a multihospital system significantly reduced LOS and time to operative fixation. An interdisciplinary HFCP can be successfully implemented across a hospital system. The program resulted in a statistically significant reduction in overall LOS and a clinically meaningful reduction in time to surgery from nonemergent admission.
